# Cancer therapy and cachexia

**DOI:** 10.1172/JCI191934

**Published:** 2025-08-01

**Authors:** Tuba Mansoor Thakir, Alice R. Wang, Amanda R. Decker-Farrell, Miriam Ferrer, Rohini N. Guin, Sam Kleeman, Llewelyn Levett, Xiang Zhao, Tobias Janowitz

**Affiliations:** 1 Cold Spring Harbor Laboratory, Cold Spring Harbor, New York, USA.; 2 Department of Pharmacological Sciences, Stony Brook University, Stony Brook, New York, USA.; 3 Medical Scientist Training Program, Stony Brook University School of Medicine, Stony Brook, New York, USA.; 4 Stony Brook University, Graduate Program in Genetics, Stony Brook, New York, USA.; 5 Northwell Health, New Hyde Park, New York, USA.

## Abstract

A central challenge in cancer therapy is the effective delivery of anticancer treatments while minimizing adverse effects on patient health. The potential dual impact of therapy is clearly illustrated in cancer-associated cachexia, a multifactorial syndrome characterized by involuntary weight loss, systemic inflammation, metabolic dysregulation, and behavioral alterations such as anorexia and apathy. While cachexia research often focuses on tumor-driven mechanisms, the literature indicates that cancer therapies themselves, particularly chemotherapies and targeted treatments, can initiate or exacerbate the biological pathways driving this syndrome. Here, we explore how therapeutic interventions intersect with the pathophysiology of cachexia, focusing on key organ systems including muscle, adipose tissue, liver, heart, and brain. We highlight examples such as therapy-induced upregulation of IL-6 and growth-differentiation factor 15, both contributing to reduced nutrient intake and a negative energy balance via brain-specific mechanisms. At the level of nutrient release and organ atrophy, chemotherapies also converge with cancer progression, for example, activating NF-κB in muscle and PKA/CREB signaling in adipose tissue. By examining how treatment timing and modality align with the natural trajectory of cancer cachexia, we underscore the importance of incorporating physiological endpoints alongside tumor-centric metrics in clinical trials. Such integrative approaches may better capture therapeutic efficacy while preserving patient well-being.

## Introduction

A critical challenge in oncology is balancing the need to achieve tumor regression while minimizing the systemic adverse consequences of cancer therapies. This challenge is compounded by the dual impact of the malignancy and its treatment, which individually and together disrupt physiological homeostasis, drive multi-organ dysfunction, and weaken overall patient health. Several considerations, including potentially life-threatening side effects of therapies, such as neutropenic sepsis or liver failure, can limit treatment and clinical trial options for patients (1). One of the less frequently considered complications is cancer-associated cachexia, perhaps because it is an imprecisely defined and complex syndrome characterized by involuntary weight loss, apathy, anorexia, skeletal muscle wasting, and profound metabolic disturbances (2). Affecting up to 80% of patients with advanced cancer and contributing to 20% of cancer-related deaths (3, 4), cachexia remains underdiagnosed in clinical practice, underreported in trials, and insufficiently addressed in treatment protocols (5, 6).

Untreated cancers usually progress to become systemic diseases, particularly in the context of metastatic progression. Beyond the direct effects of tumor invasion and tissue replacement, cancer exerts widespread influence through the release of circulating factors that disrupt normal organ function, metabolism, neuroendocrine biology, and interorgan communication (7–12). Consequently, patients frequently present with or develop weight loss, apathy, and anorexia during disease progression (13–15), symptoms and clinical signs that are similar to those associated with treatment toxicities (16). These manifestations may initially be mild but often worsen to the degree that cancer-associated cachexia is diagnosed, which is clinically defined by involuntary weight loss, often coupled with anorexia, of more than 5% over the preceding 6 months of visit (17, 18).

Despite the progress made in developing diverse anticancer therapies, the systemic effects of treatments regularly extend beyond their antitumor effect, often resulting in patient-reported complications that significantly impair quality of life and mirror the effects exerted by progressive untreated cancers (19–22). Across the major therapeutic modalities, including surgery, radiotherapy, targeted therapy, chemotherapy, and immunotherapy, patients frequently experience reduced energy levels, diminished appetite, weight loss, apathy, and cognitive decline (23–25), mirroring cachexia symptoms. Consequently, careful assessment of patient-reported outcome measures, such as lack of appetite, should be coupled with blood tests used to monitor disruptions of organ function, and radiological and biomarker-based assessments should be used to monitor tumor burden in clinical practice (26).

This perspective delineates the systemic effects of cancer and its therapies, focusing on organ-specific disruptions and the interorgan communication pathways central to cancer progression and therapy-induced toxicity. We examine the temporal and dynamic interactions among cancer biology, therapeutic interventions, and disease progression. We show that the therapeutic window for balancing antitumor efficacy with systemic harm is defined by treatment timing and intensity. We highlight converging mechanisms underlying both treatment-related toxicity and cancer-associated cachexia, with a focus on their impact on key organs, including muscle, adipose tissue, liver, brain, and heart. By integrating these perspectives, we attempt to provide a framework for understanding the interplay among cancer, therapy, and whole-body physiology for clinicians treating patients with cancer cachexia. We conclude by highlighting opportunities within clinical trials and treatment strategies to develop interventions that mitigate systemic dysfunction while maximizing therapeutic effectiveness.

## Scope and considerations

While this review integrates insights from both human studies and preclinical models, much of the mechanistic work discussed is derived from murine systems. Studying cachexia and treatment toxicity at a molecular level in humans is inherently challenging due to the limited access to tissues and the invasive nature of many mechanistic investigations. Preclinical models, particularly murine systems, provide a controlled environment to reproducibly examine the effects of cancer treatments and the pathways underlying cachexia (4). These models have been instrumental in uncovering fundamental mechanisms that inform our understanding of cancer cachexia and its systemic consequences, while also guiding the development of potential therapeutic approaches. In the sections below that refer to mechanistic work, this perspective is based on murine studies unless otherwise stated.

We selected a subset of mechanisms relevant to therapeutics taken from the 2023 World Health Organization (WHO) Model List of Essential Medicines (27) (summarized in Table 1, with a more detailed list of examples in Supplemental Table 1; supplemental material available online with this article; https://doi.org/10.1172/JCI191934DS1). We included cancer therapeutics whose adverse effects have defined mechanisms and acknowledge that other examples could have been chosen. We do not extend our work to discuss the relevance of noncancer comorbidities and their medications, aging-related progressive physiological changes (28), and sex-related physiological differences (29). However, we recognize that they are important determinants of whole-body condition and treatment tolerance. Last, in this Review, we focus on how therapies activate cancer cachexia–relevant pathways and therefore do not extend our considerations to another important aspect, namely how cancer cachexia pathways can drive tumor progression.

## Mechanisms of muscle and fat loss in cancer cachexia

Skeletal muscle and fat loss in cancer cachexia result from both shared and distinct molecular mechanisms that drive systemic energy imbalance (3). Proinflammatory cytokines, IL-6, TNF-α, and TGF-β, activate key transcription factors such as NF-κB and STAT3, promoting proteolysis in skeletal muscle via the ubiquitin/proteasome system (UPS) and enhancing lipolytic signaling in adipose tissue (30, 31). Parathyroid hormone-related protein (PTHrP) further amplifies catabolic signaling, driving UPS-mediated muscle degradation and stimulating thermogenic and lipid-catabolic activity in fat depots (32). Additionally, crosstalk between macrophages and cancer cells enhances NF-κB/STAT3 signaling, increasing tumor-derived TWEAK (TNF-like weak inducer of apoptosis), a potent inducer of muscle wasting through UPS activation (33)

Other tumor-derived factors contribute to systemic catabolism. Cancer-secreted exosomal proteins, such as HSP70 and HSP90, stimulate TLR4 and p38β MAPK pathways, exacerbating muscle breakdown (34). In adipose tissue, fat loss is driven by IL-6– and TNF-α–induced activation of lipases including hormone-sensitive lipase (HSL) and adipose triglyceride lipase (ATGL), which promote lipid mobilization and energy expenditure (35–37). Insulin resistance in the host further shifts metabolism toward catabolism, limiting nutrient storage and compounding energy deficits (38). Tumor-derived oncostatin M and zinc–α2-glycoprotein (ZAG) also promote lipid mobilization, reinforcing adipose tissue depletion (39, 40).

These interconnected pathways drive cancer cachexia, yet they also overlap with mechanisms by which cancer therapy induces systemic metabolic dysfunction. While the molecular mechanisms of cachexia have been extensively defined in numerous studies (3, 41), this Review does not aim to reiterate these well-characterized pathways. Instead, it examines the converging effects of cancer and its therapies on systemic physiology, with a focus on shared inflammatory and metabolic mechanisms that drive cachexia. We emphasize organ-specific disruptions and interorgan communication pathways, highlighting how tumor- and treatment-derived factors activate overlapping molecular cascades across muscle, adipose tissue, liver, brain, and heart. In parallel, we examine the temporal interplay between cancer biology, therapeutic interventions, and disease progression, showing how treatment timing and intensity shape the therapeutic window by balancing antitumor efficacy with systemic harm. By integrating these perspectives, we provide a framework for understanding how cancer and therapy cooperatively drive whole-body physiological decline, and where intervention opportunities to mitigate toxicity while preserving treatment effectiveness may lie.

## Adverse interorgan effects of cancer therapy and cachexia

**Figure 1:**
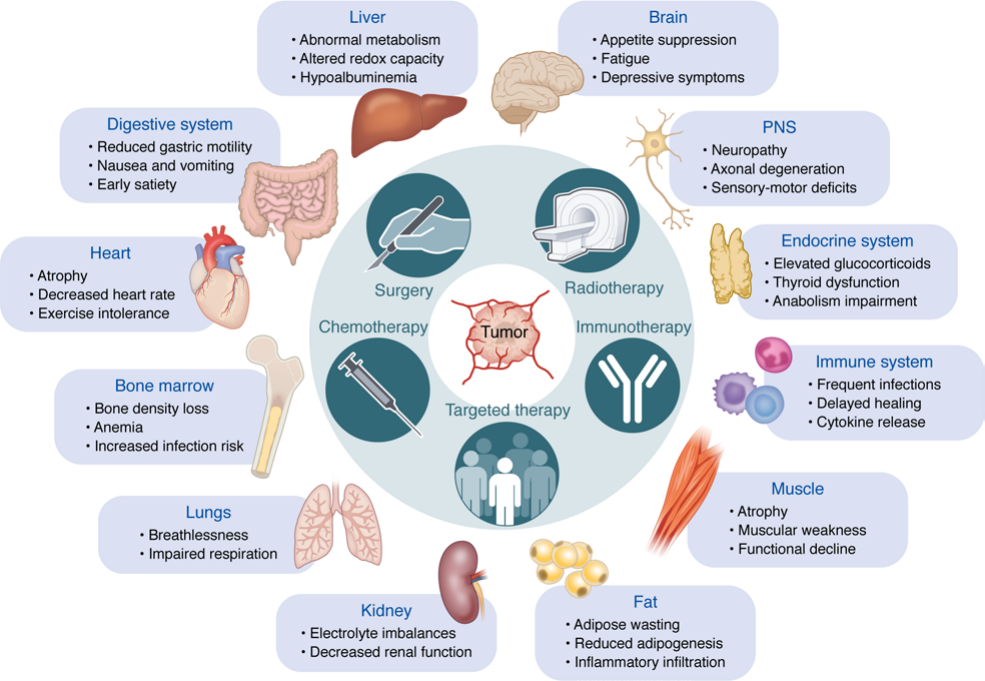
Conceptual framework: systemic interplay between cancer, therapy, and organ dysfunction in cachexia. Tumor-secreted factors lead to changes in the cellular compartments which ultimately, cause biochemical changes that may create a positive feedback loop to drive factor secretion. Cancer therapies affect cachexia development by interacting with tumors, for example, by influencing tumor-secreted factors and altering cellular and biochemical components. More specifically, the figure illustrates the interconnected systemic interactions among cancer, its treatments (surgery, chemotherapy, radiotherapy, immunotherapy, and targeted therapies), and their effects on organ function, indicating the central role of interorgan communication in patient morbidity and the development of cancer cachexia. Each organ-specific list represents a set of examples of clinically observed symptoms (e.g., breathlessness in the lungs) and underlying biochemical or pathological changes (e.g., disrupted redox balance in the liver or cytokine-driven immune dysregulation).

Understanding the physiological changes associated with cachexia and treatment toxicity requires consideration of both organ-specific impairments and interorgan interactions (Figure 1). This can be illustrated, for example, through examination of nutrient intake and processing. Here the brain plays a central role, as sensing of treatment toxicity and/or systemic inflammation suppresses appetite, drives fatigue, and induces apathy, thereby reducing caloric intake (10, 42, 43). The effects of treatment toxicity on the digestive system further compound these challenges, as chemotherapy-induced peripheral neuropathy and reduced motility exacerbate nausea, diarrhea, and impaired nutrient absorption (44, 45). Together these factors contribute to a state of malnutrition, while a loss of barrier function in various organs heightens susceptibility to systemic inflammation and infection (46). Function of the liver, a critical hub of metabolic regulation, is compromised by altered nutrient flux, redox imbalances, and diminished biosynthetic capacity, together worsening the negative energy balance, biosynthetic deficit, and metabolic stress characteristic of cachexia (47–51). This energy deficit drives fat wasting and muscle atrophy, which are not solely a consequence of nutrient deficits but may also stem from direct mechanistic drivers, in the context of both treatment toxicity and cancer progression (52–54). Adipose tissue wasting is often accompanied by inflammatory infiltration, contributing to a proinflammatory environment that perpetuates systemic dysfunction (55). In parallel, skeletal muscles experience severe atrophy, reduced regenerative capacity, and thus progressive weakness, resulting in diminished physical function leading to cachexia development (56, 57).

One consequence of this persistent metabolic and inflammatory stress, compounded by use of immune-modulating medications, is a state of immune suppression (58) that is already a risk of many chemotherapeutics and some targeted therapies due to bone marrow suppression. Coupled with immune suppression is the frequent occurrence of anemia due to chronic illness and cancer treatments, impairing the body’s ability to fight infections and reducing oxygen transport (59, 60). Anemia can lead to breathlessness, which can also result from cardiac atrophy, reduced cardiac contractility and diaphragmatic weakness, which are processes that can compound each other (61, 62). These consequences, combined with reduced renal filtration of toxic therapies due to tubular damage (63, 64), can further lead to host deterioration.

This interconnected network of effects underscores the need for integrated therapeutic strategies that address and prevent the molecular causes and consequences of treatment toxicity and cachexia. Clinicians must consider the impact of both cancer and treatments on patients to preserve organ function and physical condition and improve quality of life.

## Convergence of treatment toxicity and cachexia

**Figure 2:**
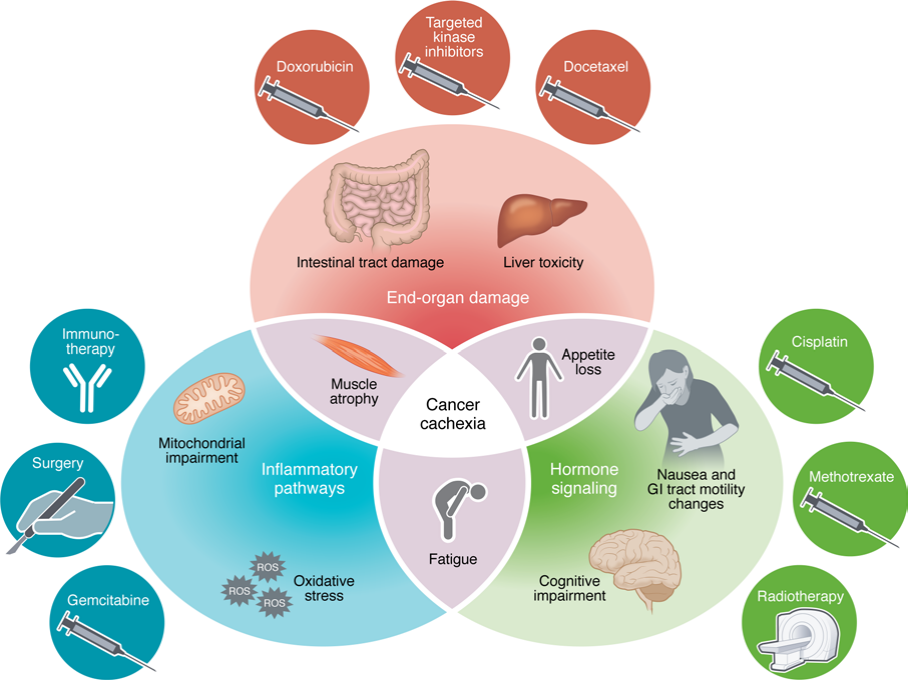
Detrimental contribution of treatment toxicity to cachexia. The interplay between inflammatory pathways, hormone signaling, end-organ damage, and patient experience (frequently reported by patients or relatives) in the intersection of progression of cancer cachexia and therapy is illustrated. Example treatments or treatment categories as well as toxicity examples are provided within each domain, demonstrating how they may contribute to systemic dysfunction and cachexia development.

Cancer cachexia and treatment toxicity arise through overlapping molecular mechanisms. We have identified three broad mechanisms of cachexia induction as a consequence of cancer progression (Figure 2): (i) Inflammatory processes can alter organ function to promote cachexia. Key cytokines such as IL-6, which can be tumor secreted, cause central and peripheral dysfunction, namely in the brain and liver, respectively. IL-6 disrupts dopaminergic motivation, resulting in apathy and fatigue (10, 43), while suppressing hepatic ketogenesis, exacerbating systemic energy imbalances (47). (ii) Hormonal signaling can alter metabolism and tissue homeostasis, resulting in negative energy balance. Growth differentiation factor 15 (GDF-15) is increased due to prolonged inflammation. It signals to the brain to activate circuits driving food aversion, thereby reducing nutrient intake and leading to a negative energy balance (65). (iii) Direct effects on end organs such as skeletal muscle and liver can lead to cachexia. For example, activin A can induce muscle degradation through upregulation of SMAD2/3 signaling (66). This pathway disrupts protein synthesis, promotes proteolysis, and ultimately leads to muscle atrophy and weakness.

These three mechanisms of cancer cachexia, inflammatory pathways, hormonal signaling, and end-organ effects, contribute distinct and convergent pathways leading to this state and often account for mechanisms by which tumor treatments inadvertently amplify systemic dysfunction. For example, chemotherapy, targeted therapies, immunotherapy, radiotherapy, and surgery can exacerbate inflammatory cytokine production, hormonal dysregulation, or catabolic signaling in different organs, thereby magnifying the metabolic and functional impairments initially driven by the tumor itself.

## Mechanisms of treatment toxicity and cachexia

**Figure 3:**
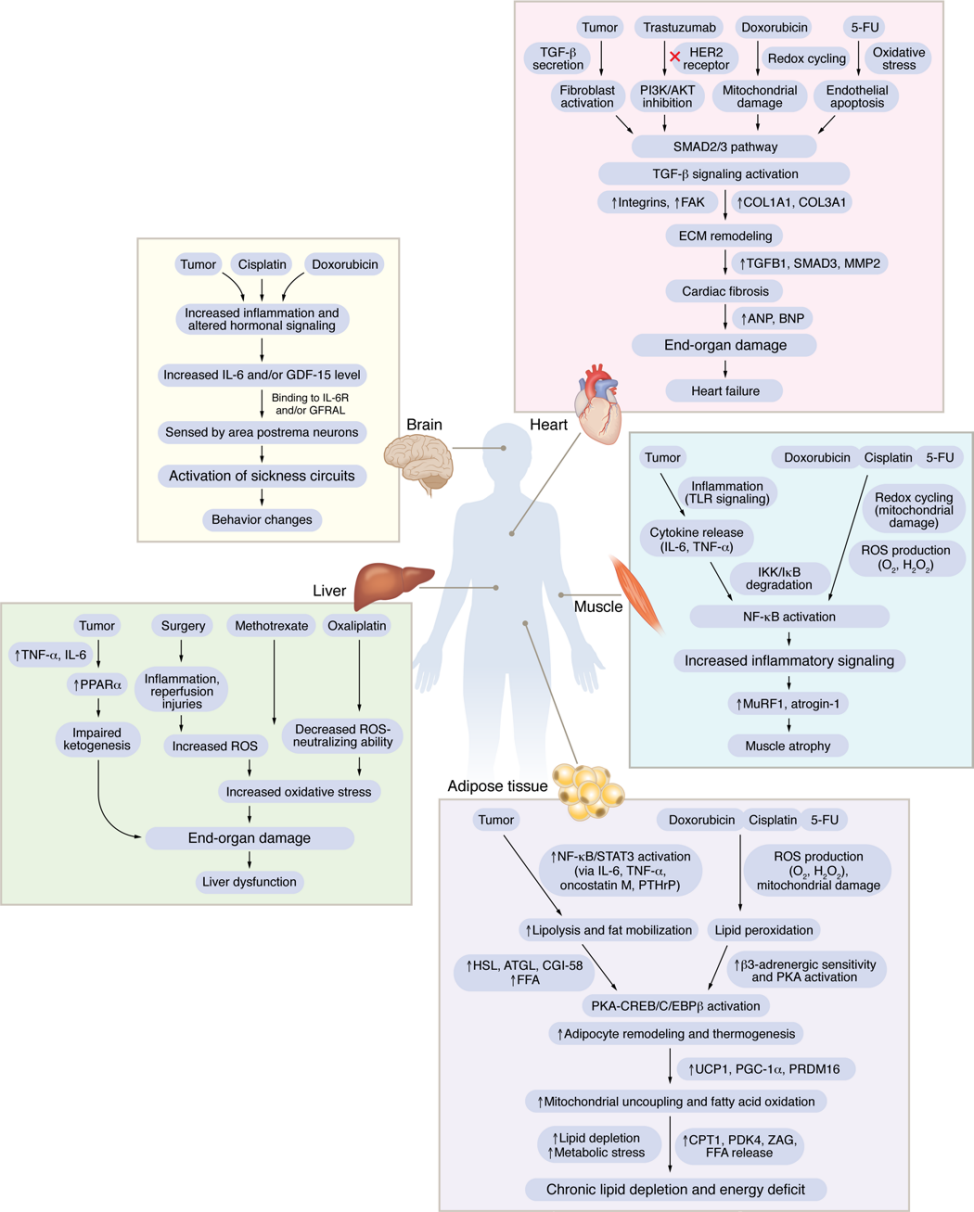
Mechanistic pathways underlying tumor- and therapy-induced cachexia across key organs. Examples of converging molecular pathways through which tumors and cancer therapies drive cachexia-associated changes in five major organ systems: muscle, liver, fat, brain, and heart. Arrows indicate the connected mechanistic pathway resulting in physiological dysfunction in each organ, ultimately leading to a convergent effect. For example, in the brain, elevated GDF-15 or IL-6 levels, resulting from tumor progression or chemotherapy, are detected by neurons in the area postrema, resulting in the activation of circuitry that leads to food avoidance and behavior changes driven by hormone signaling (42, 43, 94, 96). In the heart, tumor- and therapy-driven activation of TGF-β signaling promotes cardiac fibrosis and heart failure (181–184). In the liver, tumor- and therapy-induced ROS accelerate fibrosis and impair liver function (47, 109, 139, 185, 186). In muscle, tumors and chemotherapy agents (e.g., doxorubicin, cisplatin) activate the NF-κB axis (inflammatory pathways), leading to atrophy via upregulation of MuRF1 and atrogin-1 (187–192). In adipose tissue, lipolytic enzymes (HSL, ATGL) and β3-adrenergic/PKA/CREB signaling promote lipid mobilization and thermogenesis, leading to energy wasting and fat loss (31–37, 39, 40). These molecular pathways collectively unmask or exacerbate cachexia and contribute to multi-organ dysfunction and failure during cancer progression and therapy. The figure illustrates only selected examples and does not represent a comprehensive set of molecular pathways or causalities. ANP, atrial natriuretic peptide; BNP, brain natriuretic peptide; MMP2, matrix metallopeptidase 2; COL1A1, collagen type I alpha 1; COL3A1, collagen type III alpha 1; CGI-58, comparative gene identification-58; FFA, free fatty acid; PKA, protein kinase A; CREB, cAMP response element-binding protein; C/EBPβ, CCAAT/enhancer binding protein beta; UCP1, uncoupling protein 1; PGC-1α, peroxisome proliferator-activated receptor gamma coactivator 1-alpha; PRDM16, PR domain containing 16; CPT1, carnitine palmitoyltransferase I; PDK4, pyruvate dehydrogenase kinase 4.

Building on these foundational categories, specific molecular processes emerge that bridge the effects of cachexia and treatment toxicity. By examining circulating factors such as hormones and cytokines and then their downstream impacts on target organs, we can delineate the precise pathways through which anticancer therapies exacerbate systemic dysfunction (Figure 3). In the following sections, we examine these mechanisms according to treatment modality, chemotherapy, immunotherapy, radiotherapy, and targeted therapies, each discussed through the lens of (i) inflammatory activation, (ii) hormonal signaling, and (iii) end-organ damage.

## Chemotherapy

### Inflammatory pathways.

Doxorubicin, an anthracycline chemotherapeutic, disrupts DNA replication and triggers apoptosis, giving rise to ROS (67), which amplifies cytotoxicity, and activates NF-κB signaling and downstream production of TNF-α, IL-1β, and IL-6 (68, 69). This cytokine surge contributes to cachexia by promoting muscle protein degradation and inhibiting synthesis, manifesting as muscle wasting (70, 71). Additionally, doxorubicin-induced cardiotoxicity exacerbates cachexia by impairing cardiac function through mechanisms that involve the proteasomal degradation of TNF receptor–associated factor 2 (TRAF2), a component crucial for NF-κB signaling, ultimately promoting necrotic cell death in cardiac myocytes and worsening the systemic energy deficit (72).

5-Fluorouracil (5-FU), an antimetabolite chemotherapeutic agent, targets various cancers by inhibiting thymidylate synthase, an enzyme essential for DNA synthesis. Beyond its direct antitumor effects, 5-FU elevates proinflammatory cytokines such as TNF-α and IL-6, which are instrumental in promoting muscle wasting and cachexia (73). Preclinical studies have further elucidated 5-FU’s role in cachexia, showing changes in immune cell composition and a reduction in CD45^+^ immune cell infiltration into muscle tissues, highlighting a complex interaction between cancer pharmacotherapy and systemic muscular degeneration (74).

Gemcitabine, a nucleoside metabolic inhibitor used to treat various cancers, including pancreatic, breast, ovarian, and non–small cell lung cancer (NSCLC), functions by inhibiting ribonucleotide reductase. Gemcitabine has been observed to activate proinflammatory pathways, markedly increasing cytokines such as IL-6 and IL-8 through CD95/CD95L signaling (75). Additionally, gemcitabine is associated with serious cardiotoxic effects, such as heart tissue damage, further complicating the patient’s overall health and response to cancer treatment (76, 77).

Bleomycin is a cytotoxic chemotherapy agent known for its ability to bind to DNA and induce strand breaks through free radical generation. Bleomycin has been implicated in the promotion of cachexia through increased IL-6 and IL-33 production, which triggers lung fibrosis and muscle wasting (78).

Cyclophosphamide and ifosfamide, chemotherapeutic alkylating agents, interfere with DNA replication and RNA transcription by adding alkyl groups to DNA, leading to cell death. Cyclophosphamide induces a cytokine storm involving IL-1β, IL-7, IL-15, IL-2, IL-21, and IFN-γ, which while boosting antitumor responses also intensifies systemic inflammation that contributes to muscle wasting (79). Similarly, ifosfamide affects immune modulation by altering dendritic cell functions and increasing levels of cytokines, including IL-10, TNF-α, and IFN-γ, further impacting cachexia (80, 81). The metabolic byproducts of ifosfamide, notably 2-chloroacetaldehyde, are linked to neurotoxic effects and systemic inflammatory responses that increase cachexia risks (82, 83).

Cisplatin increases inflammation through NF-κB activation (84), IL-6 signaling, and ROS formation in neurons (85). Oxaliplatin increases the formation of neutrophil extracellular traps (NETs), which leads to mechanical hyperalgesia by inducing inflammasome release and increasing IL-18 levels (86). Paclitaxel increases IL-6, TNF-α, and CCL2 production in dorsal root ganglia neurons (87), and IL-6 neutralizing antibody pretreatment prevents peripheral neuropathy development (88), suggesting the role of increased inflammation in peripheral neuropathy development.

Methotrexate inhibits dihydrofolate reductase and influences nucleotide synthesis, which causes apoptosis in cells with high mitotic activity. Methotrexate induces appetite loss by decreasing ghrelin transportation and increasing serotonin secretion (89, 90), nausea by influencing substance P expression (91), and mucositis by interfering with mucosal cell growth (92). It also increases inflammatory markers and necrosis in the intestinal tract, which further worsens nutrient absorption (46, 93).

### Hormonal signaling.

Chemotherapy may cause fatigue, vomiting, and weight loss in patients by upregulating circulating factors such as cytokines and hormones. GDF-15, which binds to its receptor, glial cell–derived neurotrophic factor family receptor alpha-like (GFRAL), is upregulated in increased cellular stress and can lead to behavior changes such as food aversion, fatigue, and anxiety (42, 43, 94, 95). It induces fatigue in cisplatin-treated preclinical models. Cisplatin-treated mice exhibit elevated GDF-15 levels and decreased wheel-running activity, which were both prevented by administration of GFRAL-neutralizing antibodies (96). A similar effect has been demonstrated in nonhuman primates (65). There is ongoing work to evaluate the effect of GDF-15 neutralization in clinical trials (97). Moreover, cisplatin has been shown to decrease levels of plasma ghrelin, a hormone responsible for stimulating food intake and appetite, and may play a role in cancer treatment–induced dyspepsia (98, 99).

In addition to nutrient processing deficits and hormonal level changes, chemotherapy, such as 5-FU and carboplatin, and radiotherapy can increase inflammation and alter levels of the neurotransmitters serotonin, dopamine, and norepinephrine (100–102). These neurotransmitters are crucial to cognitive function, learning, memory performance, and mood regulation, which are highly relevant to daily physical function.

### End-organ damage.

As a DNA intercalating agent, doxorubicin affects both nuclear and mitochondrial DNA equally. Mitochondrial dysfunction triggers the removal of damaged organelles through autophagy, as evidenced by the upregulation of autophagy-related proteins, such as Beclin-1, autophagy-related protein 12 (ATG12), ATG7, and the microtubule-associated proteins 1A/1B light chain 3 (LC3) with an increased LC3-II to LC3-I ratio (103, 104). Imbalanced autophagy accelerates organelle degradation, protein degradation, and, ultimately, cell death in the affected muscle cells. In addition to autophagy pathways, doxorubicin triggers activation of the ubiquitin/proteasome pathway in both skeletal and cardiac muscle tissues. This activation is mediated by muscle-specific E3 ligases, such as atrogin-1 and MuRF-1, which are responsible for the polyubiquitination and subsequent degradation of muscle proteins (105). In skeletal muscle, doxorubicin induces overexpression of FoxO1 and FoxO3 transcription factors, which further amplify the transcription of genes associated with muscle atrophy and enhanced protein degradation (106). Cancer-upregulated E3 ligase UBR2 plays a critical role in cachexia by targeting the fast-twitch muscle fiber isoforms MHC II-b and II-x for proteasomal degradation, resulting in loss of contractile function in fast fibers, which contributes to cancer cachexia (107).

The alkylating agents melphalan, oxaliplatin, carboplatin, cisplatin, cyclophosphamide, and ifosfamide increase cytotoxicity through DNA crosslinking and oxidative stress, which cause cardiotoxicity, hepatotoxicity, nephrotoxicity, pulmonary toxicity, and pain hypersensitivity (108–117). In in vivo and in vitro models, decreased glutathione reductase and increased lipid peroxidation in multiple organs after alkylating agent treatment are possible explanations for organ toxicity (113, 115–118). Aside from increasing oxidative stress, cisplatin upregulates ubiquitin–proteasome–related genes such as MuRF-1 and Atrogin-1, leading to increased degradation of muscle proteins (119), which contributes to further muscle deterioration and cachexia development.

Cytotoxic agents such as bleomycin, capecitabine, docetaxel, and paclitaxel interfere with DNA synthesis or replication, eventually resulting in cell death. Docetaxel and paclitaxel increase the activity of oxidation enzymes, such as PKC and NADPH oxidase (120, 121). This upregulation coupled with decreased ROS scavenger enzyme, which neutralizes ROS, increases oxidative stress (120, 121). An increase in ROS induces liver, renal, and heart injury in in vivo models treated with cytotoxic agents (122–124).

Topoisomerase inhibitors etoposide and irinotecan inhibit DNA strand relaxing during DNA replication and transcription. The antimetabolites fluorouracil and methotrexate interfere with nucleic acid synthesis and vinca alkaloids vinblastine, and vinorelbine interferes with microtubule synthesis and disassembly, which are crucial in cell division. In in vivo and in vitro models, these agents result in increased oxidative stress in the heart, spleen, and intestine (125–132). Irinotecan can exacerbate autophagy-dependent apoptosis in cancer cells by increasing production of ROS and activating stress-related pathways such as JNK and P38 MAPK, which further promote autophagy in cancerous tissues (133).

As demonstrated through the mechanisms outlined above, chemotherapy contributes to cachexia not merely through collateral toxicity, but by activating molecular pathways that converge with those induced by cancer itself. Across diverse agents, recurring features, including NF-κB–driven cytokine surges, hormone-mediated appetite suppression, and end-organ damage via oxidative stress, highlight a shared pathophysiological landscape. This convergence between tumor- and treatment-induced dysfunction amplifies inflammation, disrupts metabolism, and accelerates physiological decline. Recognizing these overlaps clarifies how chemotherapy intensifies cachexia and reveals opportunities for targeted mitigation.

## Immunotherapy

### Inflammatory pathways.

Immune checkpoint inhibitors such as nivolumab and ipilimumab, approved for treating various cancers, inadvertently promote cachexia through their immune-modulating actions. Nivolumab blocks programmed cell death protein 1 (PD-1) interactions on T cells with programmed death ligand 1 (PD-L1) on tumor cells, and ipilimumab inhibits cytotoxic T lymphocyte–associated protein 4 (CTLA-4) to enhance T cell activation. This heightened immune response, though beneficial against tumors, also leads to increased cytokine production (134, 135). CAR T cell therapy has been a transformative development in cancer treatment; it is specifically engineered to enhance the immune system’s ability to target and destroy cancer cells by recognizing specific antigens (136). Despite its effectiveness, the therapy’s mechanism of action produces a notable complication, cytokine release syndrome (137), which is marked by the increased release of inflammatory mediators such as IL-1, IL-6, and GM-CSF. This cytokine storm induces systemic inflammatory responses that can substantially impact the patient’s metabolism and body composition. In addition to increased inflammation, high inflammatory cytokine levels could lead to neurotoxicity in the central nervous system (138). This causes further decline in physical health in patients who are at risk of cachexia development.

Although immunotherapy-induced inflammation is well documented, mechanistic evidence for its direct effects on hormonal signaling or end-organ toxicity remains limited. However, immune-mediated toxicities converge with tumor-driven mechanisms, amplifying systemic dysfunction and accelerating cachexia in patients receiving immunotherapy.

## Surgery and radiotherapy

### Inflammatory pathways.

Surgery is the first-line treatment for resectable solid tumors and is often combined with adjuvant therapies. Anesthetic agents used in surgery and the surgery procedure itself can lead to increased production of inflammatory cytokines and increased oxidative stress (139). Sevoflurane, an anesthetic agent, activates the NF-κB signaling pathway, which upregulates production of the inflammatory cytokine IL-6 (140). Patients with a history of immunotherapy may experience cytokine release syndrome during radiotherapy treatment, leading to raised IL-6 levels (141, 142).

### End-organ damage.

Radiotherapy causes DNA damage and cell-cycle arrest by delivering high-energy radiation to cells. However, similar DNA damage and mitochondrial dysfunction occur in adjacent normal tissue. Damaged tissues increase global TGF-β and collagen levels, which contributes to cardiac toxicity and fibrosis (143). Fibrosis formation in the heart can cause further decline in cardiac function and decreased physical ability (144, 145).

Although these modalities are often viewed as localized interventions, their systemic consequences, especially inflammation and fibrosis, may interact with tumor-induced stress to heighten vulnerability to cachexia.

## Targeted therapy

### Inflammatory pathways.

Trastuzumab, a targeted therapy for HER2-positive cancers, is associated with cardiotoxicity through multiple mechanisms, including mitochondrial damage and increased oxidative stress within cardiac cells (146, 147). TGF-β– and IL-6–high environments promote cardiac fibrosis and structural remodeling (148), which further impairs cardiac function and contributes to heart failure. These adverse effects are exacerbated when trastuzumab is used in conjunction with cardiotoxic agents, such as doxorubicin, ultimately leading to worsening of cardiac outcomes and increased risk of heart failure (149).

By triggering cardiotoxicity and inflammatory remodeling, targeted therapies such as trastuzumab can reinforce pathophysiological processes already initiated by the tumor, thereby exacerbating cachexia-related decline.

## Converging mechanisms across organs

Despite differences in therapeutic class, cancer treatments often activate the same inflammatory, hormonal, and metabolic pathways as the tumor itself, compounding systemic dysfunction. These shared mechanisms affect key organs, including muscle, adipose tissue, liver, brain, and heart, driving cachexia through oxidative stress, cytokine release, and disrupted energy homeostasis. The result is accelerated physiological decline and reduced treatment tolerance. Figure 3 illustrates these overlapping pathways and highlights potential targets for interventions aimed at preserving patient strength and function.

## Reversibility of cachexia drivers

There are ongoing efforts to target key mediators of cachexia in hopes of preventing progression or reversing functional decline. For example, antibodies targeting IL-6 and GDF-15 have been tested in patients with NSCLC and pancreatic cancer. In a phase II trial (ClinicalTrials.gov NCT00866970), an anti–IL-6 antibody (ALD518) was shown to be well tolerated and improve hemoglobin levels, reduce fatigue, and stabilize weight, though without a clear survival benefit. Tocilizumab was combined with gemcitabine/nab-paclitaxel in a phase II study (NCT02767557) to treat patients with advanced pancreatic cancer and demonstrated lower muscle loss compared with gemcitabine/nab-paclitaxel therapy alone (150). More recently, a GDF-15–neutralizing antibody (ponsegromab) was evaluated in a phase II trial (NCT05546476), where it increased body weight and lean mass and improved appetite and physical function, with a favorable safety profile. However, treatment response may depend on cytokine levels prior to treatment initiation (97, 151) and on route of administration as demonstrated in vivo (43). Further understanding of the mechanism of disease will improve treatment outcomes and the possibility of reversing cachexia development.

## Dynamic effects of cancer therapy and cachexia

**Figure 4:**
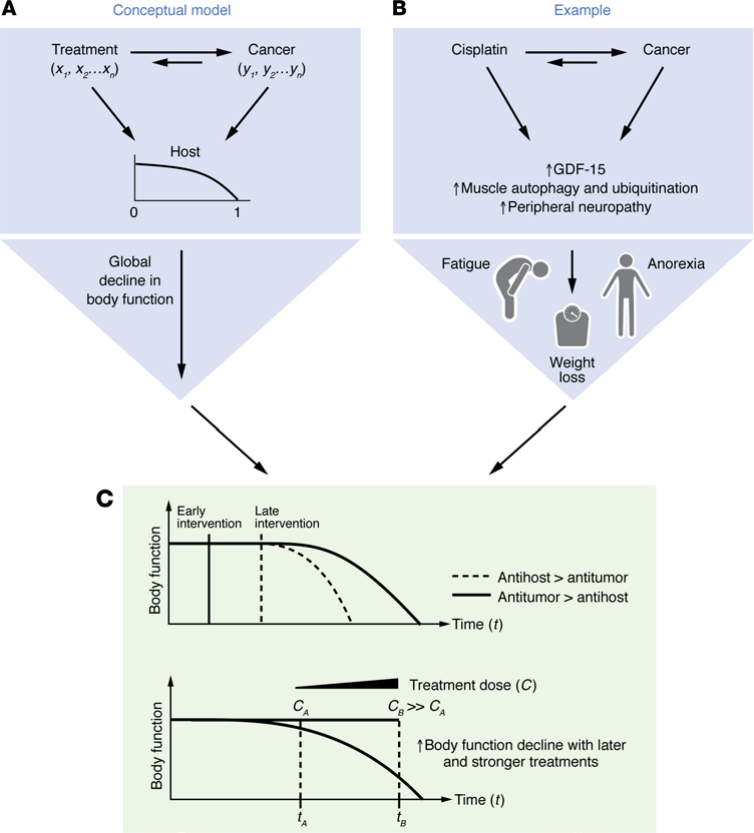
Dynamic effects of cancer treatment on outcome and cachexia. Conceptualization of the interplay between cancer treatment efficacy and toxicity (therapeutic window), disease progression, and the risk of developing cachexia. (**A**) Concept: Cancer and treatment have reciprocal interactions via factors *x_1_*, *x_2_*…*x_n_* and *y_1_*, *y_2_*…*y_n_*, and both affect the host system over time. The composite interactions determine how much the global body function declines. 0 indicates a nonsymptomatic precancerous state when body function is well preserved, and 1 indicates the end point when body function declines to a survival threshold. (**B**) Specific example: Cisplatin treatment can reduce tumor burden and consequently tumor-associated GDF-15 levels, but it can also elevate GDF-15 levels through induction of cell stress in multiple tissues and can reduce its own excretion by reducing renal filtration rates. A net increase in GDF-15 level, therefore, can increase cachexia susceptibility potentially even in the context of reduced tumor burden. (**C**) A pseudotime representation of body function shows that as body function declines, the therapeutic benefits diminish, and the same intervention may ultimately become detrimental because of the host effect. Therefore, an early intervention when body function is still preserved may maximize net benefits and promote survival. As discussed in “Scope and considerations,”we did not include covariables in this discussion but acknowledge that they may have an impact on body function and the interaction between cancer and treatments.

The effects of treatments on the cancer-bearing host are time- and dose-dependent (Figure 4). Delayed cancer treatment is related to worse treatment outcomes, and late-stage cancer treatments sometimes only offer marginal benefits or cause harm (152). This is reflected in the use of the Eastern Cooperative Oncology Group Performance Status (ECOG PS) scale, in which determination of a high value in patients indicates that initiation of burdensome therapies should be avoided. When given early in the disease trajectory, the antitumor effects of the therapies are more likely to outweigh the unwanted side effects on the host. As discussed above, many cancer therapies, while effective as an antineoplastic agent, exhibit cumulative, dose-dependent toxicities that could exacerbate cachexia (153). For example, cisplatin, while effective in tumor suppression, induces a progressive increase in GDF-15 and a decline in ghrelin levels over time, leading to appetite loss, reduced physical activity, and worsening cachexia symptoms (65). This demonstrates how temporal changes in treatment burden can shift physiological responses from resilience to vulnerability.

These dynamics extend beyond cytotoxic agents. Glucocorticoids such as dexamethasone and prednisone are frequently prescribed to manage the symptoms associated with cancer and its treatment, such as reduced appetite, chemotherapy-induced nausea, prevention of edema after irradiation of spinal cord–compressing metastases, and cerebral edema (154, 155). These steroids activate glucocorticoid receptor signaling to directly suppress inflammatory immune responses, reduce edema, and temporarily enhance patient comfort and quality of life (156). However, their use is not without challenges, as glucocorticoids suppress systemic immunity, for example, manifesting as reduced efficacy of checkpoint immunotherapy (157), and phenocopy the organ atrophy observed in cachexia. Steroid-induced muscle atrophy is driven by activation of the ubiquitin/proteasome pathway, leading to increased muscle protein degradation via specific ligases such as MuRF1 and MAFbx (158). Simultaneously, glucocorticoids inhibit protein synthesis by altering mTOR signaling and induce insulin resistance (159), which impairs nutrient uptake and utilization by muscle cells, exacerbating muscle mass loss. Last, glucocorticoids are mainly metabolized by the cytochrome P450 (CYP) 3A4 enzyme (160, 161). The activity of CYP3A4 can be modulated by medications such as tyrosine kinase inhibitors, leading to changes in drug concentration and elimination time (162). Given these factors, the use of glucocorticoids in cancer treatment requires careful consideration to ensure that their benefits outweigh the risks, a complex question in the setting of cachexia. Optimizing dosage and treatment duration can help mitigate the catabolic effects of glucocorticoids and preserve muscle mass, though it may be challenging to demonstrate this unequivocally in clinical trials.

## Considerations for clinical trials for patients with cancer

There are currently no approved therapies for the treatment of cachexia. Guidelines and recommendations for clinical management provide modest evidence supporting the use of short-term glucocorticoids and progesterone pharmacotherapy but remain largely inconclusive about dietary and nutritional recommendations. The lack of conclusive clinical trial evidence limits our ability to treat cachexia (163); however, the concepts and biology presented in this Review could help bridge this critical gap. Mechanistic insights into cachexia and its interplay with anticancer treatments underscore the importance of addressing systemic dysfunctions that impact both therapeutic efficacy and patient well-being (164). Herein, we highlight several factors that may inform the design and execution of clinical trials to optimize outcomes for all cancer patients, including those at risk of developing cachexia.

### Patient selection and stratification.

Effective clinical trials need to account for patient heterogeneity and comorbidities, particularly regarding the patients’ risk of developing cachexia. Biomarkers such as levels of inflammatory cytokines and hormones, radiological changes in organ volume, or muscle degradation markers can help identify patients at risk of cachexia or systemic dysfunction (26, 165). Stratifying patients based on these factors may ensure trials address both cancer progression and the broader impacts of treatment on host physiology.

### Trial endpoints.

Traditional endpoints such as overall survival and tumor response may be complemented by metrics that capture systemic health, including functional recovery, actimetry, and quality of life using established surveys such as the Functional Assessment of Chronic Illness Therapy (FACIT) measurement and mobile health data (166). These measures are critical for trials involving patients with or at risk of cachexia, as they provide a more complete evaluation of therapeutic efficacy and tolerability. Different trial endpoints that consider cancer stage rather than mortality could accelerate trial completion (167). In addition, early-phase clinical trials may benefit from clinical and mechanistic effect monitoring that is ideally tracked and analyzed longitudinally, perhaps using remote monitoring in combination with biological sample analysis. To identify ideal trial hypotheses and endpoints, an integration of preclinical models would provide valuable insights into the mechanisms linking cancer progression, treatment toxicity, and cachexia.

### Adaptive trial design.

Adaptive trial designs may be essential for addressing the evolving nature of cancer progression and systemic wasting. These frameworks allow prespecified changes based on interim patient responses, such as early signs of weight loss or metabolic decline (168, 169). This approach enables timely implementation of supportive strategies, including nutritional interventions, physical therapy, and pharmacologic agents. For example, a phase II clinical trial demonstrated the outcome of using technologies such as wearable devices to conduct remote clinical trials (106). Targeted therapies such as anamorelin, a ghrelin receptor agonist that stimulates appetite and lean mass gain, and ponsegromab, a monoclonal antibody that neutralizes GDF-15–mediated anorexia, may be most effective when guided by biomarker-based patient stratification (170, 171). Integrating these strategies into adaptive trial designs enhances clinical relevance and supports personalized care to preserve patients’ strength, function, and quality of life.

### Improved diagnosis and coding.

Accurate diagnosis and clinical disease coding of cachexia and its early markers in clinical settings may enhance patient identification and data collection for trials (5). This may even extend to patients at risk of developing cachexia, enabling more targeted, earlier interventions and robust analysis.

By incorporating these principles, clinical trials may better capture the interplay between tumor progression, treatment toxicity, and systemic health, ultimately improving outcomes for all patients with cancer.

## Future directions for patient-based research

The complexity of cancer cachexia necessitates high-resolution approaches to dissect its molecular and cellular drivers. Technological advances such as single-cell RNA-Seq (scRNA-Seq), single-nucleus RNA-Seq (snRNA-Seq), and spatial transcriptomics are applicable to human tissue samples and have provided unprecedented insights into transcriptional changes across immune cells, muscle fibers, and adipose tissue, uncovering key tumor-derived cytokines (IL-6, TNF-α, TWEAK, and PTHrP) and their downstream catabolic pathways (55, 172, 173). Leveraging these insights, monoclonal antibodies and bispecific molecules targeting IL-6, GDF-15, and activin A are emerging as potential interventions to suppress catabolic signaling and preserve muscle mass (174, 175). PTHrP-neutralizing therapies may reduce energy expenditure, mitigating systemic wasting (32). To advance personalized cachexia management, biomarker-driven patient stratification should be prioritized in clinical trials (176). For example, GDF-15 has been linked to appetite suppression and muscle wasting in specific cachexia subtypes and is currently under investigation as a clinical biomarker (170, 177). However, its expression varies among patients, underscoring the need to identify additional biomarkers for more precise patient selection and therapeutic targeting. High-resolution molecular profiling will be essential for refining cachexia subtypes and should be combined with careful clinical phenotyping, including detailed analyses of patient-reported outcome measures, with the aim of predicting treatment responses and guiding personalized interventions (178). Additionally, artificial intelligence–driven (AI-driven) predictive modeling and adaptive clinical trial designs will further enhance patient-specific therapeutic strategies, optimizing both survival and quality of life (179, 180).

## Conclusion

The advancement of cancer treatments requires a deep understanding of how the mode and timing of therapy, cancer biology, disease progression, physiology, and environment impact host condition and patient care. To date, research has primarily focused on antitumor effects to quantify treatment efficacy. This Review highlights the importance of also considering the host organism in cancer management, using as an example the risk of cachexia development. Combined assessments of patient-specific conditions and biological responses are essential for minimizing side effects and maximizing effectiveness. The emerging understanding of interorgan effects during systemic processes, such as cachexia, offers an avenue to improved clinical trial and care design. Emphasizing a more comprehensive approach, enabled by an ever-increasing tool set to capture biological and clinical response data, will lead to better patient outcomes, improving both survival rates and quality of life.

## Supplementary Material

Supplemental table 1

## Figures and Tables

**Table 1:**
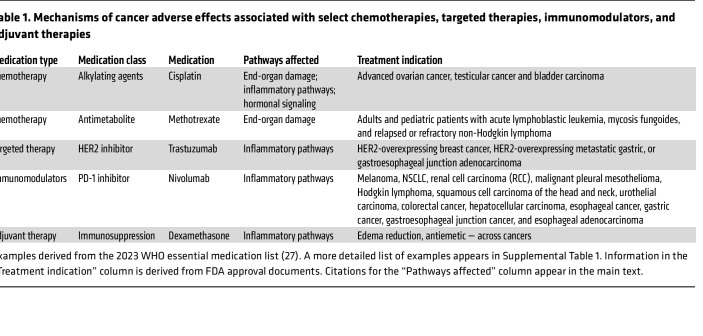
Mechanisms of cancer adverse effects associated with select chemotherapies, targeted therapies, immunomodulators, and adjuvant therapies

## Version 1 08/01/2025

Electronic publication
